# Abdominal compressions during cardiopulmonary resuscitation: a scoping review

**DOI:** 10.1590/0034-7167-2022-0400

**Published:** 2023-11-27

**Authors:** Magda Mileyde de Sousa Lima, Maria Aline Moreira Ximenes, Dariane Veríssimo de Araújo, Lívia Moreira Barros, Nelson Miguel Galindo, Joselany Áfio Caetano

**Affiliations:** I Universidade Federal do Ceará. Fortaleza, Ceará, Brazil; II Universidade da Integração Internacional da Lusofonia, Afro-Brasileira. Redenção, Ceará, Brazil.; III Instituto Federal de Educação, Ciência e Tecnologia de Pernambuco. Pernambuco, Ceará, Brazil.

**Keywords:** Heart Arrest, Cardiopulmonary Resuscitation, Return of Spontaneous Circulation, Heart Massage, Abdominal Cavity, Paro Cardiaco, Reanimación Cardiopulmonar, Retorno de la Circulación Espontánea, Masaje Cardiaco, Cavidad Abdominal, Parada Cardíaca, Reanimação Cardiopulmonar, Retorno da Circulação Espontânea, Massagem Cardíaca, Cavidade Abdominal.

## Abstract

**Objectives:**

to map the scientific evidence on the use of abdominal compressions during cardiopulmonary resuscitation in patients with cardiac arrest.

**Methods:**

this is a scoping review based on the question: “What is the evidence regarding the use of abdominal compressions during cardiopulmonary resuscitation in patients with cardiac arrest?”. Publications up to August 2022 were collected from eight databases. The Preferred Reporting Items for Systematic Reviews and Meta-Analyses extension for Scoping Reviews was used.

**Results:**

seventeen publications were included. The identified general population consisted of adults and elderly individuals. The primary outcome revealed significant rates of return of spontaneous circulation. Secondary outcomes indicated a significant improvement in heart rate, blood pressure, oxygen saturation, and other outcomes.

**Conclusions:**

abdominal compressions have been shown to be beneficial. However, further clinical studies are needed to identify the best execution method and its impacts.

## RESUMO

**Objetivos:**

mapear as evidências científicas sobre o uso de compressões abdominais durante a reanimação cardiopulmonar em pacientes com parada cardiorrespiratória.

**Métodos:**

trata-se de uma revisão de escopo, baseada na questão: “quais são as evidências sobre o uso de compressões abdominais durante a reanimação cardiopulmonar em pacientes com parada cardiorrespiratória?”. Foram coletadas as publicações até agosto de 2022 em oito bases de dados. Foi utilizado o *Preferred Reporting Items for Systematic Reviews and Meta-Analyses extension for Scoping Reviews*.

**Resultados:**

incluiu-se 17 publicações. O público geral identificado foi composto por adultos e idosos. O desfecho primário evidenciou taxas significativas de retorno da circulação espontânea. Os desfechos secundários indicaram melhora significativa na frequência cardíaca, pressão arterial, saturação de oxigênio e outros resultados.

**Conclusões:**

as compressões abdominais mostraram-se benéficas. No entanto, mais estudos clínicos são necessários para identificar o melhor método de execução e seus impactos.

## RESUMEN

**Objetivos:**

mapear la evidencia científica sobre el uso de compresiones abdominales durante la reanimación cardiopulmonar en pacientes con paro cardíaco.

**Métodos:**

esta es una revisión de alcance basada en la pregunta: “¿Cuál es la evidencia con respecto al uso de compresiones abdominales durante la reanimación cardiopulmonar en pacientes con paro cardíaco?”. Se recopilaron publicaciones hasta agosto de 2022 de ocho bases de datos. Se utilizó la extensión de Informes Preferidos para Revisiones Sistemáticas y Metaanálisis para Revisiones de Alcance.

**Resultados:**

se incluyeron diecisiete publicaciones. La población general identificada estaba compuesta por adultos y personas mayores. El resultado primario reveló tasas significativas de retorno de la circulación espontánea. Los resultados secundarios indicaron una mejora significativa en la frecuencia cardíaca, la presión arterial, la saturación de oxígeno y otros resultados.

**Conclusiones:**

las compresiones abdominales han demostrado ser beneficiosas. Sin embargo, se necesitan más estudios clínicos para identificar el mejor método de ejecución y sus impactos.

## INTRODUCTION

In recent years, alternatives to conventional manual Cardiopulmonary Resuscitation (CPR) have been applied with the aim of increasing perfusion during resuscitation attempts after cardiac arrest and improving patient survival. One of these possibilities is the inclusion of manual abdominal compressions as opposed to the rhythm of chest compressions^(1)^.

One of the techniques for abdominal compression is Interposed Abdominal Compression CPR (IAC-CPR), which requires the presence of three rescuers and involves a combination of conventional chest compression with intermittent abdominal compression. One rescuer compresses the abdomen, another compresses the chest, and the third provides ventilation^(2)^.

To correctly execute the technique, the professional responsible for abdominal compressions must compress this area at the beginning of the relaxation phase of chest compression, at an intermediate location between the xiphoid process and the navel. The hand position, depth, rhythm, and frequency of abdominal compressions are similar to chest compressions, and the required force is similar to that used for palpating the abdominal aorta^(3)^.

Studies indicate that when the described IAC-CPR technique is applied, positive effects occur in the patient’s hemodynamics during and after resuscitation, compared to standard CPR^(3-4)^. These effects include improvement in oxygen metabolism parameters and arterial blood gas levels 30 minutes after the arrest^(3)^. Additionally, it is effective in treating patients with sudden cardiac arrest who have contraindications for chest compressions^(4)^.

Considering the listed benefits of IAC-CPR, the American Heart Association Guidelines for Emergency Cardiovascular Care indicate that IAC-CPR is a useful method in CPR, provided there is sufficient trained personnel available, as late or incorrect practice can impair the entire management of cardiac arrest^(5)^.

Thus, there is evidence of benefits and a recommendation for the practice of IAC-CPR by competent bodies. However, in the guidelines, its level of evidence is categorized as Class IIb^(5)^, meaning that although not strongly recommended, IAC-CPR has more positive aspects than negatives. Its application in daily practice is limited by the need for trained and qualified professionals.

Despite the classification in the guidelines regarding the use of IAC-CPR, this study becomes relevant as it is necessary to disseminate all evidence regarding the application of this technique through scientific publications. Therefore, mapping this evidence is an advantageous option, providing healthcare professionals with an overview of the possibilities of using this technique. This can favor patient care in cardiac arrest situations that would benefit from the use of this strategy, contributing to increased survival rates for this target population.

It should be noted that, despite a systematic search in the relevant scientific literature, no publications were identified that presented a synthesis of the available evidence in the literature on the use of abdominal compressions in CPR.

## OBJECTIVES

To map the scientific evidence on the use of abdominal compressions during cardiopulmonary resuscitation in patients with cardiac arrest.

## METHODS

### Ethical Aspects

As the study did not involve human subjects, it was not submitted to the research ethics committee. It should be noted that the study adhered to the ethical and legal principles of Resolution 510/2016 of the National Health Council, which applies to research involving public domain content^(6)^.

### Study design, period, and location

A scoping review was chosen as the approach for this study, as it aims to synthesize evidence from research, categorize, or group existing literature in a specific field in terms of its nature, characteristics, and volume^(7)^. The theoretical framework of the Joanna Briggs Institute Reviewer’s Manual proposed in 2020 was used as a guide for the methodological description^(7)^. The study protocol was registered in the Open Science Framework under registration osf.io/mdafu. Data were collected from publications up to August 2022, searched during the period from July to August 2022.

### Population or sample

From the database searches, a sample of 699 publications was identified, of which 467 were excluded for not meeting the inclusion criteria, 88 for not addressing the central question, and 107 for being duplicate studies, resulting in a total of 17 articles in the final sample. The study population was determined to be patients with cardiac arrest.

### Inclusion and exclusion criteria

All published articles that addressed the research question were included, with no language or time period restrictions. Only studies with specific contexts, such as research with animals, comments, news, expert consensus, and articles not published in full, were excluded.

### Study protocol

To formulate the research question, the P-C-C strategy^(8)^ was used, where “P” represents the population/participants (patients with cardiac arrest), “C” represents the concept to be investigated (abdominal compressions), and “C” represents the context (cardiopulmonary resuscitation). The established research question was: “What is the evidence on the use of abdominal compressions during cardiopulmonary resuscitation in patients with cardiac arrest?”.

Before defining the search terms, a search was conducted in the PubMed database to identify the most frequently used descriptors and keywords in studies related to the topic of interest. Thus, the following search strategy was developed, crossing the PCC strategy terms with boolean connectors AND and OR, as described in Chart 1.

**Chart 1:** Description of the databases used and search strategy employed, Fortaleza, Ceará, Brazil, 2022

Database	Search Strategy
Scopus, Pubmed/Medline, Pubmed/PMC, Web of Science, Cinahl, Scielo, Cochrane e Embase	(“heart arrest” OR “death, sudden, cardiac” OR “cardiac arrests” OR “cardiopulmonary arrest” OR “sudden death” OR “cardiopulmonary resuscitation” OR “basic life support” OR “advanced life support” OR “fist aid” OR “basic cardiac life support” OR “cpr” OR “cardio-pulmonary resuscitation” OR “life support, basic cardiac” OR “heart massage” ) AND (“abdominal compression” OR “abdominal compressions” OR “adbomen” OR “abdominal lifting” OR “abdominal compression decompression”)

The search for studies was conducted independently by two researchers, and in case of disagreement, a third researcher made the final decision.

After the study selection, duplicate publications were identified and excluded. Subsequently, the eligibility evaluation process began, with screening of the studies through the reading of titles and abstracts. Later, the full text of the selected studies was read to confirm eligibility and ensure the inclusion of relevant studies on the subject. It is worth noting that any inconsistencies or doubts in this process were resolved through consensus and discussion among the authors and other reviewers, if necessary.

The data from the articles were independently mapped using a standardized electronic form. Data were collected on the study type, design, and country; population characteristics; setting; events, rhythm, or cause of cardiac arrest; intervention; and outcomes. Additionally, the studies were classified with a level of evidence and strength of recommendation, following Oxford recommendations^(9)^. The data were then tabulated for presentation, as appropriate.

### Analysis of results and statistics

The data were compiled and tabulated in a spreadsheet using Excel 2016, and statistical analysis, including percentage calculations, was performed for a consistent presentation of the evidence. The results were presented in the form of two tables: one providing a description of the selected articles and the other mapping the scientific evidence on the use of abdominal compressions during cardiac arrest.

The scoping review was conducted following the PRISMA extension for scoping reviews (PRISMA-ScR)^(10)^.

## RESULTS

**Figure 1:**
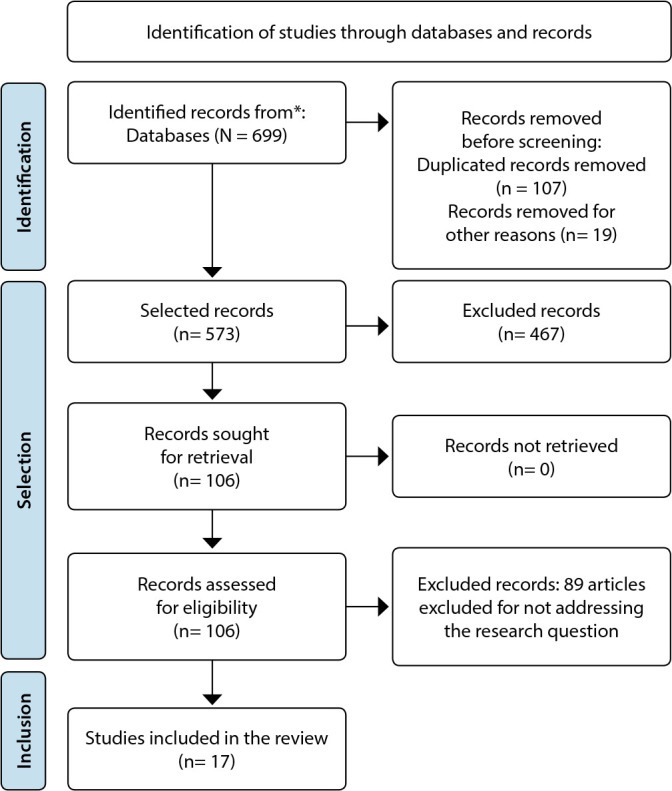
Flowchart of the search and article selection according to PRISMA 2020^(10)^, Fortaleza, Ceará, Brazil, 2022

At the end of the search, 17 articles were left, with 13 of them extracted from Pubmed/Medline, and one each from Pubmed/PMC, Scopus, Embase, and Web Of Science, respectively. The flowchart of the searches is described in Figure 1.

The articles included in the study covered the period from 1984 to 2022. Among them, seven (41.2%) were published in the 2000s, while ten (58.8%) were from the 1980s and 1990s, respectively. Regarding the origin of the publications, eight (47%) were from the United States of America (USA), three (17.6%) from China, two (11.8%) from California, two (11.8%) from Iran, and one (5.9%) each from Spain and Italy. As for the methodological approach, there was a predominance of 11 publications (64.7%) of prospective studies comparing case and control groups. All the research (100%) was conducted with adults in cardiac arrest, with an average age ranging from 54 to 85 years, as described in Chart 2.

**Chart 2:** Articles included in the scoping review listing study descriptions, Fortaleza, Ceará, Brazil, 2022

Authors/Year	Country	Design/Number of patients	Eligibility criteria	Study limitations	Outcomes	Recommendation /Evidence level
Berryman CR & Phillips GM^(11)^ (1984)	United States	Prospective study with a case group / 06 patients.	Patients in non-traumatic cardiac arrest.	Not specified.	Increased systolic and mean blood pressure with the use of abdominal compression CPR compared to standard CPR.	C/4
McDonald JL^(12)^ (1985)	United States	Prospective study with a case group / 06 patients.	Not specified	No verification of initial or mean diastolic pressure.	The technique of compressing the abdomen does not result in increased systolic blood pressures.	C/4
Mateer JR, Stueven HA, Thompson BM, et al ^(13)^ (1985)	United States	Randomized prospective study with case and control groups / 291 adults: 146 patients had standard CPR, and 145 had CPR with interposed abdominal compression.	Excluded patients with cardiac arrest due to trauma and with a history of abdominal aortic aneurysm, age <12 years, suspected pregnancy, victims of poisoning, or if intravenous access was not obtained.	Not specified.	CPR with interposed abdominal compressions did not improve resuscitation rates.	A/1B
Howard M, Carrubba C, Foss F, et al^(14)^ (1987)	United States	Prospective study with a case group / 14 adults with an average age of 56 years.	Included patients with pre-hospital cardiac arrest and excluded patients <16 years old, pregnant women, or with a history of aortic aneurysm, abdominal mass, hepato-splenomegaly, and cardiac arrest secondary to trauma.	Not specified.	The data indicate that it is unlikely to significantly improve survival rates in CPR with interposed abdominal compression.	B/2B
Ward KR, Sullivan RJ, Zelenak RR, et al^(15)^ (1989)	United States	Prospective and randomized study with case and control groups / 26 adults: 13 received standard CPR, and 13 had CPR with interposed abdominal compression.	Included patients with non-traumatic cardiac arrest and excluded those with a history of abdominal aortic aneurysm, coagulopathy, ascites, recent abdominal surgery, and pregnant women.	Low number of autopsies in non-surviving patients.	Cardiac output may be significantly increased during CPR with interposed abdominal compressions.	A/1B
Barranco F, Lesmes A, Irles JA, et al^(16)^ (1990)	Spain	Prospective study with a case group / 18 adults.	Included patients with brain death, post-anoxic encephalopathy with coma lasting more than 48 hours, and irreversible multiple organ failure.	Not specified.	The use of simultaneous chest and abdominal compressions produces higher intravascular pressure.	B/2B
Sack JB, Kesselbrenner MB & Jarrad A^(17)^ (1992)	United States	Randomized prospective study with case and control groups / 143 adults.	Included adults in cardiac arrest aged >18 years and excluded patients with traumatic cardiac arrest and a history of aortic aneurysm, recent abdominal surgery, pregnancy, and patients with endotracheal intubation taking more than 5 minutes.	Absence of neurological evaluation in the first 24 hours, inability to blind the investigators during CPR, lack of tests identifying rib fractures incidence, and lack of standardization of compressions.	The addition of interposed abdominal compression can be a useful complement to standard CPR.	A/1B
Sack JB^(18)^ (1992)	United States	Controlled and randomized trial with case and control groups / 103 adults: 55 received standard CPR, and 48 had CPR with interposed abdominal compression.	Included adults in cardiac arrest and excluded patients with a history of abdominal aortic aneurysm and suspected pregnancy.	Inability to blind the investor during the resuscitation process.	CPR with interposed abdominal compression may significantly improve survival after in-hospital cardiac arrest.	A/1B
Adams CP, Martin GB, Rivers EP, et al^(19)^ (1994)	United States	Prospective study with a case group / 20 adults.	Included normothermic adults with non-traumatic cardiac arrest in a pre-hospital setting. Patients with a history of abdominal aortic aneurysm, coagulopathy, ascites, recent thoracic or abdominal surgery, and pregnancy were excluded.	The patients received standard CPR followed by CPR with interposed abdominal compression; thus, the time may have been a bias for the abdominal CPR results. Additionally, another limitation was the non-administration of vasopressors.	CPR with interposed abdominal compression is an option during CPR as it does not require any special equipment.	B/2B
Villa GF, Colombo S, Cabrini L, et al^(20)^ (1998)	Italy	Case study / A 54-year-old man.	Not applicable	Not applicable.	Return of spontaneous circulation.	C/4
Rottenberg EM, Heard J, Hamlin R, et al^(21)^ (2011)	United States	Case study / A 56-year-old man.	Not applicable	Not applicable.	Return of spontaneous circulation.	C/4
Li J, Wang J &Li T^(22)^ (2014)	China	Prospective and observational study with case and control groups / 40 adults in cardiac arrest: 21 received standard CPR, and 19 had CPR with interposed abdominal compression.	Included patients in cardiac arrest outside the ICU and excluded arrests during the preoperative period or after the first 24 hours in the ICU.	Small size of study groups, all patients who suffered from cardiac arrest had little gastric juice in the stomach, and absence of autopsy in all non-survivors.	CPR with interposed abdominal compression after cardiac surgery is feasible and safe and may be advantageous in cardiac arrest.	B/3B
McClung C & Anshus A^(23)^ (2015)	United States	Case study / A 79-year-old woman.	Not applicable	Not applicable.	Return of spontaneous circulation.	C/4
Zhang S, Liu Q & Han S, et al^(24)^ (2016)	China	Prospective study with a case and control group / 83 adults in cardiac arrest: 43 received standard CPR, and 40 received elevation and abdominal compression CPR.	Included adults in cardiac arrest, weighing 40 to 150 kg, and excluded patients with do-not-resuscitate orders, abdominal injury, diaphragm rupture, abdominal aortic aneurysm, abdominal tumor, patients with debilitating chronic diseases, or severe tuberculosis.	Single-center study, small sample size, and absence of autopsies in non-surviving patients.	CPR with elevation and abdominal compression is associated with a higher survival rate than standard CPR.	B/3B
Movahedi A, Mirhafez SR & Behnam-Voshani H, et al^(2)^ (2016)	Iran	Randomized clinical trial with a case and control group / 80 adults.	Included adults in non-traumatic cardiac arrest, aged between 18 and 85 years, with a tracheal tube. Patients with a history of abdominal aortic aneurysm, coagulopathy, significant ascites, abdominal surgery within the last 2 weeks, active gastrointestinal bleeding, pulmonary embolism, and suspected pregnancy were excluded.	Time of cardiac arrest before CPR not estimated. Patients’ minute ventilation during CPR was not measured due to the use of bag-valve-mask device, lack of standardization of compressions, and absence of neurological evaluation within 24 hours.	CPR with interposed abdominal compression did not significantly increase the return of spontaneous circulation when compared to standard CPR.	A/1B
Li M, Song W & Ouyang Y, et al^(26)^ (2017)	China	Prospective study with a case group / 35 adults.	Included adult patients in cardiac arrest; weight between 40 and 150 kg, and with inefficient chest compressions due to chest trauma, hemothorax, or pneumothorax. Patients with abdominal trauma, diaphragm rupture, abdominal hemorrhage, abdominal aortic aneurysm, pregnancy, intestinal obstruction, abdominal cancer, ascites, or ovarian cyst were excluded.	Single-center study, small sample size, absence of a control group, and analysis of secondary outcome only after 30 minutes of venous return.	CPR with abdominal compression and elevation may improve patient outcomes.	B/2B
Khanghah AG, et al^(27)^ (2022)	Iran	Randomized Clinical Trial / 90 adults (45 in each group).	Included patients with non-traumatic cardiac arrest. Patients with hepatic cirrhosis, abdominal surgery within the last two weeks, active gastrointestinal bleeding, a history of pulmonary embolism, abdominal aortic aneurysm, significant abdominal ascites, abdominal cirrhosis, and coagulation disorders were excluded.	Absence of neurological evaluation in surviving patients.	Abdominal compression during CPR may improve resuscitation outcomes in cardiac arrest patients.	A/1B

The mapping of scientific evidence identified various mechanisms and protocols involving the use of abdominal compressions. In the primary outcome, 13 studies described the return of spontaneous circulation. Among these, nine (69.2%) reported a significant improvement in patients who received abdominal compressions. Ten secondary outcomes related to the return of spontaneous circulation were also identified. The data are detailed in Chart 3.

**Chart 3:** Mapping of scientific evidence on the use of abdominal compressions during cardiac arrest, Fortaleza, Ceará, Brazil, 2022

Management of CPR with Interposed Abdominal Compressions	
**Mechanism for performing abdominal compressions**	
Abdominal compressions are carried out using a mechanical device comprising three components: a display panel, pressure application handles, and a negative pressure device. Manual abdominal compressions.	
**Protocol 1: CPR with Elevation and Abdominal Compressio**n	
-Identification of cardiac arrest following the AHA protocol (unconscious victim, no pulse, no breathing, or agonal breathing). -Abdominal compressions are performed using the mechanical device. The depth, rate, and frequency of abdominal compressions adhere to the same guidelines as AHA-recommended chest compressions^(24-26)^. -Orotracheal intubation is conducted during advanced life support^(24)^. -The duration of compression and elevation is defined in a 1:1 ratio^(26)^. -The applied compression force is approximately 186 mmHg, and the elevation force is approximately 112 mmHg^(2)^. -The compression-to-ventilation ratio is set at 30:2^(26)^. -Medications are administered following the current AHA protocol at the time of the study^(24)^.	
**Protocol 2: CPR with Simultaneous Abdominal and Thoracic Compressions**	
-Identification of cardiac arrest following the AHA protocol (unresponsive victim, no pulse, no breathing, or gasping breathing). -The protocol was initiated 2 minutes after the cessation of aortic pressure^(16)^. -Simultaneous abdominal and thoracic compressions were performed^(16)^. -Thoracic compression was carried out using an external device with parameters in accordance with the AHA guidelines at the time. A slightly inflated blood pressure cuff was placed over the mesogastric area. A mercury column was attached to the cuff. The abdominal compressions had a pressure of 100 mmHg during the relaxation phase of the chest^(16)^.	
**Protocolo 3: CPR with Interposed Abdominal Compression**	
- Identification of cardiac arrest following the AHA protocol (unresponsive victim, no pulse, no breathing, or gasping breathing)^(2,11,13-15,17-20,22-23)^. - CPR initiation according to the AHA guidelines in effect at the time of the study^(2,11,13-15,17-19,22-23)^. - Orotracheal intubation performed during advanced life support^(13,15,17,23)^. - Defibrillation performed for shockable rhythms^(13)^. - Interposed abdominal compression during the relaxation phase of chest compression^(2,13-14,16-18,23-24)^. - The depth, rate, and frequency of abdominal compressions follow the rhythms of chest compressions indicated by the AHA at the time of the study^(17-18,22-23,25)^. - Healthcare professionals positioned their hands on the victim’s abdomen. The base of the hands was placed about 3 cm to the left of the midline, and the fingers supported gently on the abdomen. With this positioning, the abdomen was compressed using the hypothenar and thenar regions of the hands along a craniocaudal line of the abdominal aorta^(22)^. - Abdominal compressions were performed to the left of the midline to preferentially compress the abdominal aorta and minimize compression of the vena cava^(15,19-20)^. - Abdominal compressions were performed with the hands open, one on top of the other, centered over the umbilicus^(11,13,17-18)^, between the xiphoid process and the umbilicus^(24)^, or in the epigastric region^(11)^. - For intubated patients, ventilation was performed using a bag-valve-mask device^(2.17-18,21)^. - Medications were administered following the AHA protocol in effect at the time of the study^(11-12,14,17,21,23)^.	
**Primary Outcome**	**Secondary Outcome**
- Abdominal compression and elevation had a statistically significantly higher restoration of spontaneous circulation compared to the rate of chest compression [(p < 0.01)^(25)^, (p= 0.049)^(24)^]. - The return of spontaneous circulation in the group that received interposed abdominal compressions was not significantly higher than the standard CPR group [(p> 0.50)^(12,14)^, (p= 0.07)^(15)^]. - The return of spontaneous circulation in the group that received interposed abdominal compressions was significantly higher than the standard CPR group [(p> 0.05)^(22)^, (p= 0.01)^(17)^, (p= 0.007)^(18)^]. - Return of spontaneous circulation in the studied patient^(20-21,23)^.	- Significant improvement in heart rate after resuscitation with active abdominal compression and elevation [(p < 0.01)^(25)^, (p << 0.001)^(24)^, (p < 0.001)^(26)^]. - Significant improvement in blood pressure after resuscitation with active abdominal compression and elevation [(p < 0.01)^(26)^, (p = 0.003)^(24)^, (p = 0.001)^(14)^, (p < 0.05)^(12)^, (p < 0.001)^(11)^, (p = 0.003)^(28)^]. - Significant improvement in pulse oxygen saturation with active abdominal compression and elevation [(p < 0.01)^(25)^, (p < 0.001)^(24)^]. - 24-hour survival was the same in both groups [(p = 1.00)^(2)^]. - 24-hour survival was statistically higher in the group that received interposed abdominal compressions [(p = 1.00)^(17)^, (p = 0.009)^(17)^]. - CPR with interposed abdominal compression increased expired CO2 during resuscitation [(p < 0.003)^(25)^, (p = 0.001)^(15)^]. - Significantly higher hospital discharge rate in the group that received CPR with abdominal compression [(p < 0.05)^(22)^, (p = 0.02)^(18)^]. - No complications related to interposed abdominal compressions in the group of patients who underwent autopsy^(11,16-17)^. - Higher intravascular pressure during simultaneous chest and abdominal compressions^(16)^. - Regurgitation did not occur in any patients^(12-13)^.
**Indications**	**Contraindications**
- Adults aged 18 years or older with non-traumatic cardiac arrest.	-Patients with a history of abdominal aortic aneurysm, diaphragm rupture, abdominal mass, abdominal bleeding, cancer in abdominal organs, coagulopathy, hepatosplenomegaly, recent abdominal surgery, intestinal obstruction, ascites, ovarian cyst, and pregnancy or suspected pregnancy.

*AHA - American Heart Association; mmHg - Millimeters of mercury; p - significance value; CPR - Cardiopulmonary Resuscitation; CPR - Cardiopulmonary Resuscitation.*

## DISCUSSION

The analysis of the study allowed us to identify that research on the use of abdominal compression during CPR is not recent, as it dates back from 1984 to 2022. Among these, ten (62.5%) were conducted in the 1980s and 1990s. However, the American Heart Association (AHA) has not yet introduced this technique into the standard resuscitation protocol^(27)^.

In China, in 2016, the Specialized Committee of Cardiopulmonary Resuscitation of the Chinese Hospital Research Association discussed with researchers and experts the adoption of cardiopulmonary resuscitation using abdominal compressions through clinical practice^(25)^.

According to Wang et al.^(28)^, for the incorporation of abdominal compressions into CPR protocols, studies that explore the standardized and diversified methods currently available are needed. In this context, the present study identified the presence of three distinct protocols using abdominal compressions: CPR with elevation and abdominal compression, CPR with simultaneous abdominal and chest compressions, and CPR with interposed chest and abdominal compression (CTAI).

Among the identified techniques, the Brazilian Society of Cardiology, through the Guideline on Cardiopulmonary Resuscitation and Emergency Cardiovascular Care from 2013 to 2019, describes the use of auxiliary maneuvers during CPR, with CTAI standing out. According to experts, many efforts have been made by researchers in the last three decades to search for alternative techniques to standard CPR, and CTAI is a recommended technique in a hospital setting with a trained team. This alternative method involves three rescuers responsible for chest compression, abdominal compression, and ventilation, respectively. The authors emphasize that this alternative technique should take into consideration external (place of care) and internal (clinical condition of the patient) aspects^(29-30)^.

Such information corroborates with the results of the present study since the research conducted, so far, contraindicated patients with a history of abdominal diseases or suspected pregnancy. Due to the risk of worsening the clinical condition, it is unethical to include these participants in the studies, as both national and international guidelines and regulatory norms for research involving humans recommend that the study benefits should outweigh the risks^(31-32)^.

Furthermore, the relevance of implementing alternative techniques to standard CPR is highlighted because, according to Chinese researchers, the usual technique is not effective for patients with rib fractures, chest deformity, or clinical conditions of hemothorax and pneumothorax^(26)^. A study conducted in Germany with 22,565 trauma patients identified that the spontaneous circulation return rate was present in only 11% of patients who received standard CPR^(33)^.

Thus, it is believed that the use of abdominal compressions may be an alternative option to standard CPR for adults aged 18 years and older with non-traumatic cardiac arrest. It is noteworthy that the mapping conducted identified that eight studies reported the return of spontaneous circulation in patients who received abdominal compressions, while three did not identify a statistical difference when compared to patients who received only standard CPR.

In addition to the return of spontaneous circulation, the present study identified the presence of secondary outcomes: significant improvement in heart rate, blood pressure, oxygen saturation, 24-hour survival after resuscitation, increased expired carbon dioxide (CO2), higher hospital discharge rate, and absence of vomiting.

The reasons for the improvement in patients’ clinical conditions were described in 2016 by researchers from Iran. According to the authors, 20% of the blood volume is located in the abdomen. Rhythmic compressions can result in increased venous return and cardiac preload. Additionally, during the diastolic phase of chest compressions, coronary perfusion occurs. Thus, abdominal compressions during the diastolic phase increase blood flow to the heart^(1)^.

Similarly, the CTAI technique was compared using a concentrated parameter mathematical model in patients with a single ventricle shunt, which corresponds to a congenital heart disease of unique physiology, in which it is extremely difficult to resuscitate due to severe hypoxemia during CPR. Thus, Stromberg et al. (2022)^(34)^ evidenced that the use of this technique contributes to increased pulmonary blood flow, cardiac output, blood pressure, coronary perfusion pressure, and coronary blood flow, compared to standard CPR.

Another benefit evidenced in the application of CTAI would be the reduction in the incidence of gastric inflation^(35)^, which could result in a decrease in rare complications of pneumoperitoneum that lead to gastric perforation, where most require laparotomy for correction. The aforementioned complication occurs due to the accumulation of air in the abdominal cavity, as a consequence of inadequate airway management and inadequate technique of conventional CPR compressions^(36-37)^. Thus, there is growing evidence of positive outcomes regarding the effectiveness and usability of CTAI, although it remains limited by its technical difficulty^(35)^.

These data emphasize the need for studies that assess the best abdominal compression technique since the studies conducted so far have reported different abdominal compression techniques, including compressions performed to the left of the midline to preferentially compress the abdominal aorta and minimize compression of the vena cava^(15,19-20)^, and abdominal compressions performed with open hands, one over the other, centered over the umbilicus^(11,13,17-18)^, between the xiphoid and the umbilicus^(2)^, or in the epigastric region^(12)^.

In addition to the different abdominal compression techniques, it is necessary to identify the most effective force during resuscitation since the lack of standardization of the pressure exerted by the rescuers’ hands was one of the limitations of the studies conducted so far^(17,2)^. These data reinforce the need for new studies that contribute to the elaboration of a standard protocol for the use of abdominal compressions during cardiac arrest, in order to favor the improvement in the quality of care provided during this clinical emergency episode.

### Study Limitations

A limitation of the study is the exclusion of articles not published in full. This might exclude more recent research that is not yet fully available in the literature and could present new results regarding the use of abdominal compressions during CPR.

### Contributions to Nursing, Healthcare, or Public Policy

The present study provides benefits to the scientific community, nurses, and other healthcare professionals. The mapping of the main scientific evidence allowed for identifying the state of the art in using abdominal compressions during CPR. It includes information on the mechanisms of abdominal compression, the studied protocols, primary and secondary outcomes of applying this technique, indications, and contraindications, as well as eligibility criteria and the main limitations of the conducted studies.

This mapping serves as a scientific basis for recognizing this technique as an alternative to standard CPR in patients with a favorable clinical condition. Moreover, it supports the conduction of new studies aiming to explore the scarce information in the scientific literature.

## CONCLUSIONS

The majority of the studies identified that the return of spontaneous circulation in the group that received abdominal compressions is significantly higher than in the standard CPR group. Regarding secondary outcomes, the publications indicated significant improvements in heart rate, blood pressure, oxygen saturation, 24-hour survival after resuscitation, increased expired CO2, higher hospital discharge rate, and absence of regurgitation. Additionally, the studies that analyzed the bodies of non-surviving patients found no injuries in the abdominal organs.

None of the researchers contraindicated this technique. However, despite the findings, three studies did not find a statistical difference when comparing standard CPR with CPR with abdominal compression.

Finally, there is a need for new randomized clinical trials that evaluate the most effective protocol for CPR with abdominal compression and the best techniques for compressing the abdomen. Additionally, new studies should try to address the limitations of the already published studies, thus conducting research to evaluate long-term survival rates and performing autopsies on the bodies of non-surviving patients becomes necessary.
